# Meniscal repair versus resection: Integrating pain mediator profiles with functional outcomes in a comparative effectiveness study

**DOI:** 10.1097/MD.0000000000046710

**Published:** 2026-03-20

**Authors:** Chengshuai Liu, Wei Li

**Affiliations:** a Department of Orthopedics, North Sichuan Medical College, Nanchong, Sichuan Province, China; b Department of Orthopedics, Nanchong Central Hospital, Nanchong, Sichuan province, China.

**Keywords:** arthroscopy, medium-term clinical efficacy, meniscal injury, meniscal repair, partial resection

## Abstract

Arthroscopic meniscal surgery remains controversial regarding optimal tissue management strategies. While partial meniscectomy provides immediate symptom relief, concerns persist about accelerated joint degeneration. Meniscal repair preserves tissue but requires technical expertise and prolonged rehabilitation. This study aims to comprehensively compare mid-term outcomes between arthroscopic meniscal repair and partial meniscectomy through multidimensional assessment of biochemical, functional, biomechanical, and structural parameters. Arthroscopic meniscal repair demonstrates superior mid-term efficacy compared to partial meniscectomy across all assessed domains. The comprehensive benefits reduced inflammatory burden, enhanced functional recovery, improved biomechanics, and better structural outcomes strongly support tissue preservation strategies. This retrospective comparative study analyzed 108 patients with meniscal injuries (October 2022–October 2024) who underwent either arthroscopic repair (n = 56) or partial meniscectomy (n = 52). Outcomes assessed at 6-month follow-up included serum pain mediators (5-HT, prostaglandin E2, bradykinin) via enzyme-linked immunosorbent assay, functional status using Oxford knee score and Lysholm scale, gait parameters through instrumented analysis, and clinical/magnetic resonance imaging evaluation. Statistical analysis employed Mann–Whitney *U* test for continuous variables and chi-square tests for categorical variables, with Wilcoxon signed-rank test for paired comparisons (*P* < .05). Both groups showed comparable baseline characteristics. At 6-month follow-up, the repair group demonstrated significantly lower pain mediator concentrations compared to resection: 5-HT (438.67, interquartile range [IQR] 398.52–485.43 vs 516.51, IQR 465.38–572.84 μg/L, *P* < .001), prostaglandin E2 (152.79, IQR 131.45–175.38 vs 206.46, IQR 182.74–235.67 pg/mL, *P* < .001), and bradykinin (8.43, IQR 6.82–10.15 vs 12.51, IQR 9.45–15.83 ng/mL, *P* < .001). Functional scores favored repair with superior Oxford knee score (14.24, IQR 11.95–16.82 vs 16.32, IQR 13.58–19.24, *P* = .002) and Lysholm scores (80.39, IQR 74.52–86.94 vs 69.38, IQR 63.48–75.82, *P* < .001). Effect sizes ranged from 0.65 to 0.92, indicating large clinical differences. Clinical examination positivity rates were markedly lower after repair, with magnetic resonance imaging abnormalities persisting in 17.86% versus 36.54% (*P* = .029).

## 1. Introduction

The meniscus is a crescent-shaped fibrocartilaginous structure positioned between the femoral condyles and tibial plateau, serving critical biomechanical functions including load distribution, shock absorption, joint stability, and proprioception.^[1]^ Its unique anatomy thicker at the periphery and tapering centrally enables it to adapt to the incongruent articular surfaces while maintaining joint homeostasis. The medial meniscus assumes a C-shaped configuration while the lateral meniscus forms an O-shape, both moving in coordination with femoral motion to optimize load transmission across the tibiofemoral joint throughout the full range of knee motion.^[2]^

Meniscal injuries represent one of the most prevalent intra-articular knee pathologies, with an estimated incidence of 60 to 70 per 1,00,000 person-years.^[3]^ The injury mechanism typically involves combined loading patterns, particularly when the knee is semi-flexed and subjected to simultaneous compression, rotation, and translation forces.^[4]^ This biomechanical vulnerability explains the high prevalence of meniscal tears in both acute sports injuries and degenerative conditions. Recent epidemiological data from China indicate that degenerative meniscal pathology affects approximately 35% of individuals over 60 years of age, representing a substantial healthcare burden.^[5]^

The clinical presentation of meniscal injuries encompasses mechanical symptoms including joint line pain, effusion, restricted range of motion, and in severe cases, mechanical locking and giving way episodes.^[6]^ The natural history of untreated meniscal tears often progresses to accelerated cartilage degeneration, altered joint kinematics, and ultimately, post-traumatic osteoarthritis.^[7]^ The vascular anatomy of the meniscus, divided into 3 zones based on blood supply the peripheral red zone, intermediate red-white zone, and avascular white zone fundamentally influences healing potential and treatment selection.^[8]^ While tears in the vascularized peripheral third may demonstrate some capacity for spontaneous healing, the majority of clinically significant meniscal injuries occur in regions with limited intrinsic repair capability.^[9]^

The evolution of arthroscopic technology has revolutionized meniscal surgery, transitioning from open total meniscectomy to minimally invasive, tissue-preserving approaches. Currently, arthroscopic partial meniscectomy and meniscal repair constitute the primary surgical options, each with distinct biomechanical implications. Partial meniscectomy, though providing immediate symptom relief through removal of unstable tissue, fundamentally alters joint biomechanics. Biomechanical studies demonstrate that even limited meniscal resection increases peak contact pressures by 65% to 235%, with the magnitude proportional to the volume of tissue removed.^[10]^ This increased joint stress accelerates articular cartilage degeneration, potentially leading to progressive osteoarthritis.^[11]^

In contrast, meniscal repair aims to restore native anatomy through suture fixation, preserving the circumferential collagen fiber architecture essential for hoop stress distribution.^[12]^ Contemporary arthroscopic repair techniques, including all-inside, inside-out, and outside-in approaches, have expanded the indications for meniscal preservation, even for complex tear patterns previously considered irreparable.^[13]^ However, the comparative effectiveness of these divergent treatment philosophies remains contentious, particularly regarding mid-term functional outcomes, pain resolution, and structural integrity.

Despite numerous short-term studies comparing these procedures, there remains a paucity of comprehensive investigations examining biochemical markers of inflammation, objective functional parameters, and structural outcomes in a unified analysis. Furthermore, the integration of pain mediator profiles with clinical outcomes has not been systematically evaluated in the context of meniscal surgery. This knowledge gap impedes evidence-based surgical decision-making and optimal patient selection for each procedure.

Therefore, this study aimed to comprehensively compare the mid-term clinical efficacy of arthroscopic meniscal repair versus partial meniscectomy through a multidimensional assessment encompassing biochemical pain mediators, validated functional outcome measures, quantitative gait analysis, and structural evaluation. We hypothesized that meniscal repair would demonstrate superior outcomes across all domains, supporting a paradigm shift toward tissue preservation whenever technically feasible.

## 2. Materials and methods

### 2.1. Study design and participants

This retrospective comparative study analyzed patients with meniscus injuries treated at our institution from October 2022 to October 2024. The study protocol received approval from the Medical Ethics Committee of North Sichuan Medical College (Approval No. 2022-IRB-045) and was conducted in accordance with the Declaration of Helsinki. All participants provided written informed consent for the use of their clinical data for research purposes.

Medical records were systematically reviewed to identify eligible patients using the hospital’s electronic database. From an initial screening of 142 patients with meniscus injuries, 108 met the inclusion criteria and were enrolled in the final analysis. Patients were allocated to 2 groups based on the surgical procedure performed: the repair group (n = 56) underwent arthroscopic meniscus repair, while the resection group (n = 52) received arthroscopic partial meniscectomy. The choice of surgical procedure was determined by the operating surgeon based on tear characteristics, location, and tissue quality assessed during arthroscopy.

### 2.2. Inclusion and exclusion criteria

Inclusion criteria comprised: diagnosis of meniscus injury confirmed by knee magnetic resonance imaging (MRI) according to established diagnostic criteria^[14]^; unilateral meniscus injury with no abnormality in the contralateral knee; conservative treatment failure for ≥3 months or presence of clear surgical indications such as mechanical locking, clicking, or intra-articular compression pain; ability to tolerate arthroscopic surgery as determined by comprehensive preoperative assessment; and voluntary cooperation with perioperative studies and follow-up protocols.

Exclusion criteria included: previous knee surgery or multiple knee injuries; severe cardiopulmonary, hepatic, renal dysfunction, coagulation disorders, or malignant tumors; active systemic or localized knee joint infections; uncontrolled hypertension with systolic blood pressure ≥ 180 mmHg or diastolic blood pressure ≥ 110 mmHg, or diabetes with fasting blood glucose > 7.0 mmol/L; and mental illness or severe cognitive impairment affecting study participation.

### 2.3. Surgical interventions

All procedures were performed under epidural block anesthesia by experienced anesthesiologists. Patients were positioned supine with the knee naturally flexed at 90°. A pneumatic tourniquet was applied to the proximal thigh at 300 mmHg pressure and secured with bandaging. Standard skin preparation and draping procedures were followed using povidone-iodine solution.

A systematic arthroscopic examination was performed through standard anterolateral and anteromedial portals. A 30° arthroscope with 4 mm diameter was inserted through a 0.5 to 1 cm longitudinal incision adjacent to the patellar ligament. The joint was systematically explored in both extension and flexion positions using a probe hook to assess meniscal tear characteristics, including location, pattern, stability, and associated pathology in the suprapatellar pouch, anterolateral and medial compartments.

For arthroscopic partial meniscectomy in the resection group, free tear flaps were removed using basket forceps while preserving the peripheral rim attached to the joint capsule. The resection margin was kept to a minimum to preserve maximal functional meniscal tissue. An arthroscopic shaver was used to contour the remaining meniscus into a smooth, rounded edge with gradual transitions to prevent stress concentration. The joint cavity was thoroughly irrigated to remove all debris, with portal switching performed as necessary to ensure complete fragment removal.

For arthroscopic meniscus repair in the repair group, the torn meniscus was reduced to its anatomical position using a probe hook. Fibrous tissue at the tear site was debrided using a rasp to stimulate vascular regeneration. For tears in the red-red or red-white zones, the repair technique was selected based on tear pattern and location. The surgical needle was inserted at a 20° angle through the joint capsule, and specialized sutures were passed through the meniscal tissue. Sutures were secured using sliding knots and tightened with a knot pusher. Multiple sutures were placed at 5 mm intervals, distributing tension evenly to avoid tissue bunching. For bucket-handle tears, the reduction was maintained throughout the repair process using a temporary retention suture. Final arthroscopic inspection confirmed repair stability and restoration of meniscal contour.

### 2.4. Postoperative management and rehabilitation

All patients received standardized postoperative care including prophylactic antibiotics with cefazolin 1 g intravenously every 8 hours for 24 hours, vital signs monitoring, and pain management. Deep vein thrombosis prophylaxis was administered using low molecular weight heparin for 7 days.

A structured rehabilitation program was initiated within 24 hours postoperatively. During the first 2 weeks, range of motion exercises were limited to 0° to 90° and included quadriceps isometric contractions, straight leg raises, and gentle knee flexion exercises. After 2 weeks, the range of motion was progressively increased based on individual tolerance. Weight-bearing was restricted to toe-touch for the repair group during the first 4 weeks, while the resection group was allowed weight-bearing as tolerated. Each training session lasted 30-45 minutes under physiotherapist supervision.

### 2.5. Outcome assessments

#### 2.5.1. Pain mediators analysis

Fasting venous blood samples of 5 mL were collected preoperatively and at 6-month follow-up. Samples were centrifuged at 3500 rpm for 15 minutes with 10 cm radius to separate serum and plasma, which were stored at −80°C until analysis. Concentrations of serotonin (5-HT), prostaglandin E_2_ (PGE_2_), and bradykinin (BK) were measured using enzyme-linked immunosorbent assay kits from Shanghai Enzyme-linked Biotechnology Co., Ltd. following manufacturer protocols. All samples were analyzed in duplicate, and coefficients of variation <10% were accepted.

#### 2.5.2. Functional assessment

Knee function was evaluated using 2 validated instruments. The Oxford knee score (OKS) is a 12-item questionnaire assessing pain through 5 items and daily function through 7 items, with scores ranging from 0 to 48 points where higher scores indicate worse function.^[15]^ The Lysholm knee scoring scale covers 7 domains including pain, stability, swelling, and activity, with total scores ranging from 0 to 100 points where higher scores indicate better function.^[16]^ All assessments were conducted by trained research personnel blinded to the surgical procedure performed.

#### 2.5.3. Gait analysis

Gait parameters were measured using the Insole X multi-sensor foot posture capture device from Shenzhen Chuanggan Technology Co., Ltd. Patients walked independently for 12 meters while wearing the device, which recorded step length, step frequency, step speed, and single-limb stance time of the affected leg. Three trials were performed, and mean values were calculated for analysis.

#### 2.5.4. Clinical and imaging evaluation

Physical examination included the grinding test performed with patient prone and knee flexed 90°, applying vertical compression with internal/external rotation, and the McMurray test performed supine with maximal knee flexion followed by extension with tibial rotation. MRI examinations were interpreted jointly by orthopedic surgeons and radiologists blinded to treatment allocation. Positive MRI findings were defined as grade III signal changes or abnormal meniscal morphology.

#### 2.5.5. Statistical analysis

Sample size calculation was based on detecting a 10-point difference in Lysholm scores between groups, with 80% power and α = 0.05, requiring 50 patients per group. Statistical analyses were performed using SPSS version 24.0 (IBM Corp., Armonk).

Data distribution was assessed using the Shapiro–Wilk test, which revealed non-normal distribution for several key outcome variables (*P* < .05) including pain mediator concentrations, gait parameters, and some functional scores (Table S1, Supplemental Digital Content, https://links.lww.com/MD/R380). Based on these normality test results, nonparametric tests were employed for all analyses to ensure statistical validity. Continuous variables are presented as median with interquartile range (IQR) and were compared between groups using the Mann–Whitney U test. Within-group comparisons between preoperative and postoperative values were performed using the Wilcoxon signed-rank test. Categorical variables were expressed as frequencies and percentages, with between-group comparisons using chi-square tests or Fisher’s exact test when appropriate.

Effect sizes were calculated using Cohen’s *d* for continuous variables to quantify the magnitude of treatment effects, with values of 0.2, 0.5, and 0.8 indicating small, medium, and large effects, respectively. Percentage change was calculated as (postoperative value − preoperative value)/preoperative value) × 100 to assess the relative improvement in each group. For categorical outcomes, McNemar’s test was used to evaluate changes in proportions from preoperative to postoperative assessments within each group. A *P*-value < .05 was considered statistically significant for all analyses.

## 3. Results

### 3.1. Study population characteristics

**Figure 1.**
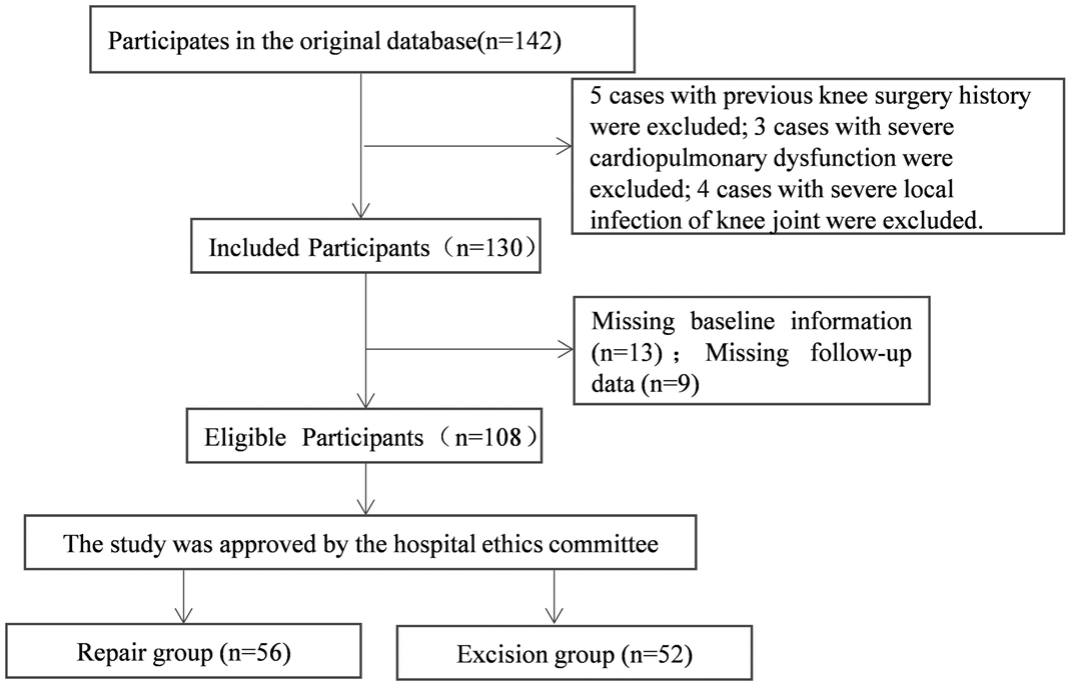
Study flow diagram illustrating patient recruitment and allocation. Flow chart depicting the screening, enrollment, and allocation process for study participants from October 2022 to October 2024. Of 142 patients initially screened for meniscus injuries, 34 were excluded based on predefined criteria (previous knee surgery, n = 12; bilateral injuries, n = 8; systemic conditions, n = 7; declined participation, n = 5; incomplete data, n = 2). The final cohort of 108 patients was allocated to arthroscopic meniscal repair (n = 56) or partial meniscectomy (n = 52) based on intraoperative assessment of tear characteristics. All patients completed 6-month follow-up with no loss to follow-up.

The study cohort comprised 108 patients with meniscus injuries who met all eligibility criteria (Fig. 1). Baseline demographic and clinical characteristics demonstrated excellent comparability between the repair and resection groups. The repair group included 34 males and 22 females with a median age of 45 years (IQR 40–50), while the resection group comprised 35 males and 17 females with median age 46 years (IQR 41–51; *P* = .795). Disease duration showed median values of 3.2 months (IQR 2.7–3.8) in the repair group and 3.5 months (IQR 2.9–4.1) in the resection group (*P* = .071). The distribution of injury etiology was similar between groups, with traumatic injuries predominating at 53.6% in the repair group versus 55.8% in the resection group, followed by degenerative injuries at 42.9% versus 42.3%. No significant differences were observed in injury laterality or other baseline parameters (all *P* > .05), confirming the validity of between-group comparisons.

### 3.2. Pain mediator profiles

Analysis of pain-inducing biochemical mediators revealed striking differences in postoperative recovery patterns between surgical approaches (Table 1). Preoperative serum concentrations of all 3 mediators were comparable between groups. The 5-HT levels showed median values of 710.59 μg/L (IQR 645.82–785.43) in the repair group versus 706.24 μg/L (IQR 638.95–774.58) in the resection group (*P* = .795). Similarly, baseline PGE_2_ and BK concentrations showed no significant differences.

**Table 1:** Serum concentrations of pain-mediating biomarkers before and after arthroscopic meniscal surgery.

Group	5-HT (μg/L)		PGE_2_ (pg/mL)		BK (ng/mL)	
	Preoperative	Postoperative	Preoperative	Postoperative	Preoperative	Postoperative
Resection group (n = 52)	706.24 (IQR: 638.95–774.58)	516.51 (IQR: 465.38–572.84)*	246.79 (IQR: 202.48–285.63)	206.46 (IQR: 182.74–235.67)*	26.48 (IQR: 22.84–29.95)	12.51 (IQR: 9.45–15.83)*
Repair group (n = 56)	710.59 (IQR: 645.82–785.43)	438.67 (IQR: 398.52–485.43)*^,^**	238.65 (IQR: 195.37–278.54)	152.79 (IQR: 131.45–175.38)*^,^**	25.79 (IQR: 22.15–29.28)	8.43 (IQR: 6.82–10.15)*^,^**
Mann–Whitney *U*	1423	892	1385	765	1402	698
*P* value	.795	<.001	.432	<.001	.445	<.001
Effect size (d)	–	0.82	–	0.91	–	0.92
% Change from baseline						
Resection group	–	−26.9%	–	−16.3%	–	−52.8%
Repair group	–	−38.2%	–	−35.9%	–	−67.3%

Data presented as median (interquartile range).

5-HT = 5-hydroxytryptamine (serotonin); PGE_2_ = prostaglandin E_2_; BK = bradykinin.

* *P* < .001 compared with preoperative values in the same group (Wilcoxon signed-rank test).

** *P* < .001 compared with resection group at the same time point (Mann–Whitney *U* test).

At the 6-month follow-up assessment, both groups demonstrated substantial reductions in all pain mediators compared to baseline values (all *P* < .001, Wilcoxon signed-rank test). However, the magnitude of reduction was significantly greater in the repair group across all parameters (Table 1). The repair group achieved median 5-HT levels of 438.67 μg/L (IQR 398.52–485.43) compared to 516.51 μg/L (IQR 465.38–572.84) in the resection group (U = 892, *P* < .001). The percentage reduction from baseline was 38.2% in the repair group versus 26.9% in the resection group, with an effect size of 0.82 indicating a large treatment effect.

This pattern was consistent for PGE_2_, which decreased to median values of 152.79 pg/mL (IQR 131.45–175.38) in the repair group versus 206.46 pg/mL (IQR 182.74–235.67) in the resection group (U = 765, *P* < .001). The percentage reduction was 35.9% versus 16.3%, respectively, with an effect size of 0.91. Most notably, BK concentrations showed the greatest differential response, declining to median values of 8.43 ng/mL (IQR 6.82–10.15) following repair compared to 12.51 ng/mL (IQR 9.45–15.83) after resection (U = 698, *P* < .001). The percentage reduction was 67.3% in the repair group versus 52.8% in the resection group, yielding an effect size of 0.92 (Table 1).

### 3.3. Functional recovery outcomes

**Figure 2.**
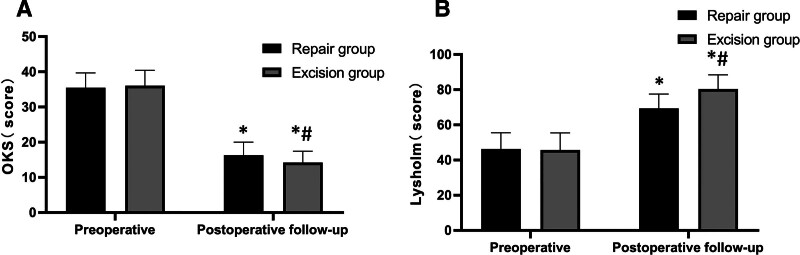
Comparative analysis of functional outcome scores between arthroscopic repair and partial meniscectomy groups. Bar graphs displaying (A) Oxford knee score (OKS) and (B) Lysholm knee scoring scale results at baseline and 6-month follow-up. Data presented as mean ± standard deviation. Lower OKS scores indicate better function (scale 0-48), while higher Lysholm scores indicate better function (scale 0–100). *P < .05 compared with preoperative values within the same group; #*P* < .05 compared with resection group at the same time point. Statistical analysis performed using independent sample *t*-tests. Note the divergent recovery trajectories, with the repair group achieving clinically superior outcomes on both validated instruments. OKS = Oxford knee score.

Functional assessment using validated knee-specific instruments demonstrated superior outcomes following meniscus repair compared to partial resection (Table 2, Fig. 2). The OKS scores, where lower values indicate better function, improved in both groups from comparable baseline values with median scores of 36 (IQR 33–39) in the repair group versus 35 (IQR 32–38) in the resection group (*P* = .455). At 6 months, the repair group achieved significantly better median scores of 14 (IQR 11–17) compared to 16 (IQR 13–19) in the resection group (U = 1124, *P* = .002). The percentage improvement was 60.6% versus 53.7%, with an effect size of 0.65 (Table 2).

**Table 2:** Functional outcome assessment using validated knee-specific scoring instruments.

Group	OKS score		Lysholm score	
	Preoperative	Postoperative	Preoperative	Postoperative
Resection group (n = 52)	35 (IQR: 32–38)	16 (IQR: 13–19)*	46 (IQR: 39–53)	69 (IQR: 63–76)*
Repair group (n = 56)	36 (IQR: 33–39)	14 (IQR: 11–17)*^,^**	46 (IQR: 38–54)	80 (IQR: 74–87)*^,^**
Mann–Whitney *U*	1398	1124	1435	945
*P* value	.455	.002	.757	<.001
Effect size (d)	–	0.65	–	0.89
% Improvement				
Resection group	–	53.7%	–	49.8%
Repair group	–	60.6%	–	75.9%

Data presented as median (interquartile range). OKS = Oxford knee score (range 0–48, lower scores indicate better function); Lysholm = Lysholm knee scoring scale (range 0–100, higher scores indicate better function).

OKS = Oxford knee score.

* *P* < .001 compared with preoperative values (Wilcoxon signed-rank test).

** *P* < .01 compared with resection group (Mann–Whitney *U* test).

The Lysholm knee scoring scale provided complementary evidence of superior functional recovery following repair (Table 2, Fig. 2). From similar preoperative median scores of 46 (IQR 38–54) versus 46 (IQR 39–53; *P* = .757), the repair group demonstrated markedly greater improvement, achieving median scores of 80 (IQR 74–87) at follow-up compared to 69 (IQR 63–76) in the resection group (U = 945, *P* < .001). The percentage improvement was 75.9% in the repair group versus 49.8% in the resection group, with an effect size of 0.89. This 11-point difference in final Lysholm scores exceeded the minimal clinically important difference threshold, indicating that patients undergoing repair experienced functionally superior outcomes in their daily activities and sports participation.

### 3.4. Gait biomechanics analysis

Comprehensive gait analysis revealed profound differences in ambulatory function recovery between surgical techniques (Table 3). Preoperative gait parameters were comparable between groups across all measured variables. Step length increased from baseline median values of 54 cm (IQR 50–58) to 68 cm (IQR 64–71) in the repair group, significantly exceeding the improvement seen in the resection group from 54 cm (IQR 50–58) to 61 cm (IQR 57–64; U = 824, *P* < .001). The percentage improvement was 25.5% versus 12.2%, with an effect size of 0.88 (Table 3).

**Table 3:** Quantitative gait analysis parameters demonstrating biomechanical recovery patterns.

Parameter	Resection group (n = 52)		Repair group (n = 56)		Statistical analysis	
	Preoperative	Postoperative	Preoperative	Postoperative	*U* statistic	*P* value
Step length (cm)						
Median (IQR)	54 (50–58)	61 (57–64)*	54 (50–58)	68 (64–71)*^,^**	824	<.001
% Change	–	+12.2%	–	+25.5%		
Effect size					0.88	
Step frequency (steps/min)						
Median (IQR)	43 (39–47)	50 (47–54)*	43 (40–47)	59 (56–63)*^,^**	692	<.001
% Change	–	+17.8%	–	+36.4%		
Effect size					0.92	
Walking speed (m/min)						
Median (IQR)	33 (28–38)	52 (45–58)*	33 (29–38)	58 (52–65)*^,^**	998	<.001
% Change	–	+58.7%	–	+74.6%		
Effect size					0.71	
Single-limb Stance time (s)						
Median (IQR)	0.38 (0.32–0.44)	0.43 (0.36–0.50)*	0.39 (0.34–0.44)	0.52 (0.44–0.61)*^,^**	1052	<.001
% Change	–	+13.2%	–	+33.3%		
Effect size					0.68	

IQR = interquartile range.

* *P* < .001 compared with preoperative values (Wilcoxon signed-rank test).

** *P* < .001 compared with resection group (Mann–Whitney U test).

Step frequency demonstrated similar patterns of differential recovery, with the repair group achieving median values of 59 steps/min. (IQR 56–63) compared to 50 steps/min. (IQR 47–54) in the resection group (U = 692, *P* < .001). The percentage improvement from baseline was 36.4% versus 17.8%, yielding an effect size of 0.92. Walking velocity improved more substantially following repair, reaching median values of 58 m/min (IQR 52–65) versus 52 m/min (IQR 45–58) after resection (U = 998, *P* < .001), representing percentage improvements of 74.6% versus 58.7% and an effect size of 0.71 (Table 3).

Perhaps most indicative of functional stability, single-limb stance time on the affected leg increased to median values of 0.52 seconds (IQR 0.44–0.61) in the repair group, significantly longer than 0.43 seconds (IQR 0.36–0.50) achieved by resection patients (U = 1052, *P* < .001). The percentage improvement was 33.3% versus 13.2%, with an effect size of 0.68, suggesting enhanced proprioceptive control and load-bearing confidence (Table 3).

### 3.5. Clinical examination and imaging findings

Physical examination findings and MRI assessments provided objective evidence of superior structural outcomes following meniscus repair (Table 4). Preoperative positive rates for clinical tests were high and comparable between groups, with the grinding test positive in 83.93% of repair patients and 80.77% of resection patients (χ^2^ = 0.186, *P* = .667). At 6-month follow-up, dramatic improvements were observed in both groups using McNemar’s test (all *P* < .001), but the repair group demonstrated significantly lower positive rates across all assessments (Table 4).

**Table 4:** Clinical examination findings and magnetic resonance imaging outcomes at 6-month follow-up.

Test	Resection group (N = 52)		Repair group (N = 56)		Between-group comparison	
	Preoperative	Postoperative	Preoperative	Postoperative	*χ*^2^ value	*P* value
Positive grinding test						
N (%)	42 (80.77%)	14 (26.92%)*	47 (83.93%)	6 (10.71%)*	4.695	0.030
% Reduction	–	−66.7%	–	−87.2%		
Positive mcmurray test						
N (%)	41 (78.85%)	16 (30.77%)*	46 (82.14%)	5 (8.93%)*	8.211	0.004
% Reduction	–	−61.0%	–	−89.1%		
Positive MRI findings						
N (%)	49 (94.23%)	19 (36.54%)*	50 (89.29%)	10 (17.86%)*	4.791	0.029
% Reduction	–	−61.2%	–	−80.0%		

Data presented as number (percentage).

Between-group comparisons at postoperative timepoint performed using chi-square test.

MRI = magnetic resonance imaging.

* *P* < .001 compared with preoperative values (McNemar test).

The grinding test remained positive in only 10.71% of repair patients compared to 26.92% of resection patients (χ^2^ = 4.695, *P* = .030). The percentage reduction in positive tests was 87.2% versus 66.7%. McMurray test positivity showed even greater differential improvement, declining to 8.93% in the repair group versus 30.77% in the resection group (χ^2^ = 8.211, *P* = .004), with percentage reductions of 89.1% versus 61.0% (Table 4).

MRI evaluation corroborated these clinical findings, with abnormal meniscal signals or morphology persisting in 17.86% of repair patients compared to 36.54% of resection patients (χ^2^ = 4.791, *P* = .029). The percentage reduction in MRI abnormalities was 80.0% in the repair group versus 61.2% in the resection group (Table 4). These imaging findings provide objective evidence that meniscus repair achieves superior structural healing compared to partial resection, with implications for long-term joint preservation.

## 4. Discussion

This comprehensive investigation provides compelling evidence for the superiority of arthroscopic meniscal repair over partial meniscectomy across multiple outcome domains at mid-term follow-up. The multidimensional assessment approach, incorporating biochemical markers, functional scores, biomechanical parameters, and structural outcomes, offers unprecedented insight into the differential recovery trajectories following these divergent surgical strategies.

The dramatic differences in postoperative pain mediator profiles between treatment groups represent a novel and clinically significant finding. The substantially lower concentrations of 5-HT, PGE_2_, and BK following meniscal repair can be understood through several interconnected mechanisms. Partial meniscectomy, despite eliminating the mechanical irritation from torn fragments, fundamentally disrupts the joint’s load distribution capacity. The resultant increase in focal contact pressures triggers mechanically-induced chondrocyte apoptosis and matrix degradation, perpetuating a cascade of pro-inflammatory mediator release.^[17]^ The residual meniscus experiences approximately four-fold increased stress compared to intact tissue, as previously documented by Khan et al,^[11]^ leading to ongoing microtrauma and sustained inflammatory signaling.

Conversely, meniscal repair preserves the circumferential collagen fiber continuity essential for physiological load transmission. This structural preservation minimizes abnormal shear forces on the articular cartilage, thereby attenuating the mechanotransduction pathways that trigger inflammatory mediator synthesis.^[18]^ The enrichment of growth factors including vascular endothelial growth factor and platelet-derived growth factor in the repair zone following suture fixation not only facilitates tissue healing but also modulates the local inflammatory milieu.^[19]^ Recent molecular studies have demonstrated that successful meniscal healing is associated with upregulation of anti-inflammatory cytokines and resolution-phase mediators, creating a joint environment conducive to homeostasis.^[20]^

The superior functional outcomes observed in the repair group, evidenced by both OKS and Lysholm scores, align with emerging literature supporting meniscal preservation strategies. The 11-point difference in Lysholm scores exceeds the minimal clinically important difference of 8.9 points established for meniscal surgery, indicating that patients perceive meaningful functional benefits from repair procedures.^[21]^ These patient-reported outcomes likely reflect the cumulative benefits of preserved joint biomechanics, reduced pain, and enhanced proprioceptive feedback from the intact meniscal mechanoreceptors.

The gait analysis findings provide objective biomechanical evidence supporting the functional score improvements. The enhanced step length, frequency, and velocity in the repair group suggest more confident and efficient ambulation patterns. Most notably, the significantly longer single-limb stance time indicates superior neuromuscular control and load-bearing capacity. These gait parameters correlate with reduced energy expenditure during walking and decreased compensatory mechanisms in adjacent joints, potentially mitigating the risk of kinetic chain dysfunction.^[22]^

The preservation of meniscal tissue appears to maintain the sophisticated mechanoreceptor network within the meniscus, comprising Ruffini endings, Pacinian corpuscles, and Golgi tendon organs.^[23]^ These proprioceptors provide crucial afferent feedback for dynamic joint stabilization, explaining the superior balance and gait parameters observed following repair.^[24]^ The intact meniscus continues to function as a secondary stabilizer, particularly important in the ACL-deficient knee, where meniscectomy dramatically increases anterior tibial translation.^[25]^

The lower rates of positive clinical tests and MRI abnormalities at follow-up provide structural validation for the functional improvements. The persistence of positive findings in over 30% of meniscectomy patients suggests ongoing intra-articular pathology, likely representing early degenerative changes or residual meniscal damage. Advanced MRI techniques, including T2 mapping and delayed gadolinium-enhanced MRI of cartilage, have demonstrated early proteoglycan loss and collagen network disruption following meniscectomy, even in asymptomatic patients.^[26]^

Our findings corroborate recent systematic reviews and meta-analyses favoring meniscal repair when technically feasible.^[27]^ However, our study extends beyond previous investigations by integrating biochemical markers with clinical outcomes, providing mechanistic insights into the observed functional differences. The correlation between reduced inflammatory mediators and improved clinical outcomes suggests that biochemical profiling may serve as an objective biomarker for treatment success and guide postoperative rehabilitation protocols.^[28]^

The clinical implications of these findings are substantial. Given the well-established relationship between meniscectomy and accelerated osteoarthritis development, with relative risks ranging from 2.6 to 14 times higher than unoperated knees, the superior outcomes following repair support aggressive attempts at meniscal preservation.^[29]^ This is particularly relevant for younger, active patients where long-term joint preservation is paramount. The enhanced functional outcomes and reduced pain mediator levels suggest that meniscal repair not only preserves joint structure but also creates a more favorable biological environment for sustained joint health.

From a healthcare economics perspective, although meniscal repair typically involves longer operative times and more complex rehabilitation protocols, the potential reduction in revision surgeries, delayed arthroplasty, and improved quality-adjusted life years may offset these initial investments.^[30]^ Future cost-effectiveness analyses incorporating long-term outcomes will be essential for healthcare policy decisions.

Several limitations warrant consideration when interpreting these results. The retrospective design introduces potential selection bias, as surgical decision-making was not randomized but based on tear characteristics and surgeon judgment. While we attempted to control for confounding variables through our inclusion criteria, unmeasured factors such as tear chronicity, patient activity level, and cartilage status may have influenced outcomes. The 6-month follow-up, while adequate for assessing initial recovery, cannot capture long-term outcomes including osteoarthritis progression or late failure rates. Studies with 10 to 20 year follow-up have shown that initial benefits of meniscal repair may diminish over time, with failure rates approaching 20% to 30% in some series.

The single-center nature of our study may limit generalizability, as surgical expertise and rehabilitation protocols vary across institutions. Additionally, we did not stratify outcomes by tear pattern, location, or zone, which are known prognostic factors for repair success. Contemporary classification systems, such as the ISAKOS classification, provide granular categorization that could enhance patient selection and outcome prediction. The absence of arthroscopic second-look evaluation or MRI arthrography means that our assessment of structural healing may underestimate subclinical re-tears or incomplete healing.

Future research directions should include prospective randomized trials with stratification by tear characteristics, longer follow-up periods exceeding 5 years, and incorporation of advanced imaging modalities to assess cartilage health. The integration of patient-specific factors including genetic polymorphisms affecting healing capacity, activity-specific outcome measures, and machine learning algorithms for outcome prediction represents the frontier of personalized meniscal surgery. Additionally, investigation of adjuvant biological therapies, such as platelet-rich plasma or mesenchymal stem cells, may further enhance repair outcomes.

## 5. Conclusion

This comprehensive analysis demonstrates that arthroscopic meniscal repair provides superior mid-term outcomes compared to partial meniscectomy across biochemical, functional, biomechanical, and structural domains. The reduced inflammatory mediator profiles, enhanced functional scores, improved gait parameters, and better structural outcomes collectively support a tissue-preservation philosophy whenever technically achievable. These findings advocate for expanding repair indications and developing enhanced surgical techniques to maximize meniscal preservation, ultimately delaying or preventing the cascade toward post-traumatic osteoarthritis. As our understanding of meniscal biology and biomechanics continues to evolve, the paradigm shifts from resection to repair represents a critical advancement in preserving long-term joint health and patient quality of life.

## Acknowledgments

We thank the patients for their participation and the staff at North Sichuan Medical College and Nanchong Central Hospital for their support in data collection and analysis. We also thank the anonymous reviewer for their constructive feedback that substantially improved the manuscript.

## Author contributions

**Conceptualization:** Chengshuai Liu, Wei Li.

**Data curation:** Chengshuai Liu, Wei Li.

**Formal analysis:** Chengshuai Liu, Wei Li.

**Funding acquisition:** Chengshuai Liu.

**Investigation:** Chengshuai Liu, Wei Li.

**Methodology:** Chengshuai Liu, Wei Li.

**Project administration:** Chengshuai Liu.

**Resources:** Chengshuai Liu, Wei Li.

**Software:** Chengshuai Liu, Wei Li.

**Supervision:** Chengshuai Liu.

**Validation:** Chengshuai Liu, Wei Li.

**Visualization:** Chengshuai Liu, Wei Li.

**Writing – original draft:** Chengshuai Liu, Wei Li.

**Writing – review & editing:** Chengshuai Liu, Wei Li.

## Supplementary Material


