# Supplementary material for: Meniscal repair versus resection: Integrating pain mediator profiles with functional outcomes in a comparative effectiveness study
Source: Medicine (Baltimore). 2026 Mar 20;105(12):e46710. doi: 10.1097/MD.0000000000046710 (PMC13008203; doi:10.1097/MD.0000000000046710)
Supplement: Supplementary file 1 [file medi-105-e46710-s001.docx]

**Supplementary Table 1. Shapiro-Wilk Test Results for Normality Assessment.**

| **Variable** | **Repair Group** | | **Resection Group** | |
| --- | --- | --- | --- | --- |
|  | **W statistic** | **P value** | **W statistic** | **P value** |
| **Pain Mediators (Postoperative)** |  |  |  |  |
| 5-HT | 0.892 | 0.012 | 0.904 | 0.028 |
| PGE₂ | 0.913 | 0.034 | 0.897 | 0.019 |
| Bradykinin | 0.878 | 0.008 | 0.885 | 0.011 |
| **Functional Scores (Postoperative)** |  |  |  |  |
| OKS | 0.945 | 0.082 | 0.952 | 0.124 |
| Lysholm | 0.958 | 0.156 | 0.949 | 0.098 |
| **Gait Parameters (Postoperative)** |  |  |  |  |
| Step Length | 0.923 | 0.042 | 0.918 | 0.035 |
| Step Frequency | 0.905 | 0.024 | 0.911 | 0.031 |
| Walking Speed | 0.897 | 0.018 | 0.902 | 0.022 |
| Stance Time | 0.884 | 0.009 | 0.891 | 0.013 |

*Note:* P<0.05 indicates significant deviation from normal distribution, justifying the use of non-parametric statistical tests
